# Kinetics of the Lactate to Albumin Ratio in New Onset Sepsis: Prognostic Implications

**DOI:** 10.3390/diagnostics14171988

**Published:** 2024-09-08

**Authors:** Irene Karampela, Dimitris Kounatidis, Natalia G. Vallianou, Fotis Panagopoulos, Dimitrios Tsilingiris, Maria Dalamaga

**Affiliations:** 1 Second Department of Critical Care, Attikon General University Hospital, Medical School, National and Kapodistrian University of Athens, 1 Rimini St., Haidari, 12462 Athens, Greece; eikaras1@gmail.com; 2 Department of Biological Chemistry, Medical School, National and Kapodistrian University of Athens, 75 Mikras Asias St., Goudi, 11527 Athens, Greece; madalamaga@med.uoa.gr; 3 Diabetes Center, First Department of Propaedeutic Internal Medicine, Laiko General Hospital, Medical School, National and Kapodistrian University of Athens, 11527 Athens, Greece; dimitriskounatidis82@outlook.com; 4 Department of Internal Medicine, Sismanogleio General Hospital, 1 Sismanogleiou St., 15126 Athens, Greece; fotis_1992@hotmail.com; 5 First Department of Internal Medicine, University Hospital of Alexandroupolis, Democritus University of Thrace, 68100 Alexandroupolis, Greece; tsilingirisd@gmail.com

**Keywords:** albumin, critical illness, kinetics, lactate, lactate to albumin ratio, mortality, outcome, prognosis, sepsis, septic

## Abstract

The lactate to albumin ratio (LAR) has been associated with the severity and outcome of critical illness and sepsis. However, there are no studies on the kinetics of the LAR during the early phase of sepsis. Therefore, we aimed to investigate the LAR and its kinetics in critically ill patients with new onset sepsis regarding the severity and outcome of sepsis. We prospectively enrolled 102 patients with sepsis or septic shock within 48 h from diagnosis. LARs were recorded at inclusion in the study and one week later. Patients were followed for 28 days. LAR was significantly lower one week after enrollment compared to baseline in all patients (*p* < 0.001). LARs were significantly higher in patients with septic shock and in nonsurvivors compared to patients with sepsis and survivors, respectively, both at inclusion (*p* < 0.001, *p* < 0.001) and at one week later (*p* < 0.001, *p* < 0.001). LARs at baseline were positively associated with the severity of sepsis (APACHE II: r = 0.29, *p* = 0.003; SOFA: r = 0.33, *p* < 0.001) and inflammatory biomarkers, such as C-reactive protein (r = 0.29, *p* < 0.1), procalcitonin (r = 0.47, *p* < 0.001), interleukin 6 (r = 0.28, *p* = 0.005) interleukin 10 (r = 0.3, *p* = 0.002) and suPAR (r = 0.28, *p* = 0.004). In addition, a higher LAR, but not its kinetics, was an independent predictor of 28-day mortality (at inclusion: HR 2.27, 95% C.I. 1.01–5.09, *p* = 0.04; one week later: HR: 4.29, 95% C.I. 1.71–10.78, *p* = 0.002). In conclusion, the LAR may be a valuable prognostic indicator in critically ill patients with sepsis at admission and one week later.

## 1. Introduction

Sepsis, a life-threatening organ dysfunction caused by a dysregulated host response to infection, constitutes a significant cause of death [1]. The estimated incidence of sepsis worldwide was 48.9 million in 2017, while 11 million deaths due to sepsis were reported, comprising nearly 20% of deaths globally [2]. The increasing prevalence of sepsis in the community as well as in hospitalized patients emphasizes the need for increased awareness, early diagnosis, and timely treatment in order to restrain the enormous burden of sepsis [3,4]. The current diagnostic criteria for sepsis and septic shock are based on the sequential organ failure assessment score (SOFA), incorporating clinical signs as well as biomarkers of organ dysfunction [1]. However, currently used biomarkers (C-reactive protein and procalcitonin) lack specificity, while the clinical use of novel biomarkers derived from recent studies remains limited [5]. Therefore, ongoing research on diagnostic and prognostic biomarkers of sepsis focuses on the combination of various biomarkers that are routinely used in clinical practice [6]. 

Among biomarkers of organ dysfunction, elevated lactate indicates impaired tissue oxygenation leading to anaerobic glycolysis. During sepsis, decreased oxygen delivery and impaired oxidative phosphorylation may be present, not only due to tissue hypoperfusion during shock but also due to microcirculatory and mitochondrial dysfunction leading to impaired tissue oxygen extraction and cellular hypoxia [7]. However, other factors such as accelerated aerobic glycolysis and impaired hepatic lactate clearance also contribute to hyperlactatemia in sepsis [8]. Lactate is closely associated with sepsis severity and mortality, and it is a clinically useful biomarker for the risk stratification of sepsis [9,10]. Noteworthy, the recent consensus definition for septic shock was based on hypotension along with elevated lactate [1].

Albumin, the most abundant protein in plasma, is a major contributor to oncotic pressure, representing the most important protein carrier in plasma, which maintains acid–base homeostasis and acts as a protein reserve. Moreover, albumin exerts antioxidant, immune, and anticoagulant actions through the inhibition of platelet aggregation and binding to antithrombin [11]. Albumin is exclusively synthesized in the liver and represents a long-term biomarker of nutrition. However, albumin is also a negative acute-phase reactant during critical illness. Albumin levels acutely decrease during sepsis due to decreased liver synthesis as a response to inflammatory molecules as well as due to catabolism driven by the higher protein and energy requirements [12]. Additionally, liver dysfunction during sepsis may decrease albumin synthesis, while increased microvascular permeability may drive albumin into extravascular compartments, contributing further to hypoalbuminemia [12,13]. Low albumin has been linked to higher morbidity and is an independent predictor of poor outcomes in hospitalized patients [14,15,16]. Furthermore, albumin is strongly associated with prognosis in patients with sepsis [17,18].

Recent research has focused on the evaluation of the predictive value of the combination of the above biomarkers expressed as a ratio, namely the lactate to albumin ratio (LAR), in critical illness, severe infections, and acute inflammation, with promising results [19,20,21]. Recent studies support a prognostic role of the LAR in sepsis [22,23]. However, there are no studies on the kinetics of the LAR during the early phase of sepsis and its association with the severity and outcome of sepsis. We hypothesized that the LAR and its kinetics, combining two routinely measured biomarkers in critically ill patients, which reflect key metabolic changes during sepsis, may be a useful prognostic tool in clinical practice. Therefore, we aimed to prospectively investigate the LAR and its kinetics in new onset sepsis in critically ill patients with regard to sepsis outcome.

## 2. Materials and Methods

### 2.1. Study Population and Protocol

In this prospective observational study, consecutive adult patients with new onset sepsis were recruited among critically ill patients hospitalized in the Intensive Care Unit (ICU) of a tertiary hospital of the Medical School of the University of Athens over a two-year period. This study conformed to the guidelines of the Declaration of Helsinki. Ethical approval was obtained by the Scientific and Ethics Committee of the hospital (reference no.: 587/10-04-2013). We obtained informed consent from all patients or their next of kin. The study protocol has been previously published [24,25,26,27,28].

All patients fulfilled the diagnostic criteria of sepsis or septic shock established by the Third International Consensus Definitions (SEPSIS-3) [1]. According to these criteria, a suspected or proven infection and an acute increase in SOFA score of ≥2 points establish the diagnosis of sepsis. Among patients with sepsis, the presence of persistent hypotension, requiring vasopressors to maintain a mean arterial pressure ≥ 65 mmHg, and a serum lactate level >2 mmol/L despite adequate volume resuscitation establish the diagnosis of septic shock. The inclusion criteria were an age ≥18 years and a diagnosis of sepsis or septic shock during the last 48 h. The exclusion criteria were malignancy, immunosuppression, liver disease, endocrine disease, total parenteral nutrition, administration of albumin- or lactate-containing fluids, and pregnancy. Demographic, clinical, and routine laboratory data were recorded. Patients hospitalized in the ICU for less than a week were excluded from the analysis. Finally, patients were followed for 28 days from enrollment, and the outcome was recorded.

### 2.2. Laboratory Analysis

We collected arterial blood samples for lactate determination as well as whole blood samples for routine laboratory investigations. Blood samples were collected from the patients upon inclusion in the study as well as one week later. Lactate was determined in arterial whole blood samples within 15 min from blood sampling in an arterial blood gas analyzer using a heparinized syringe (Cobas B 123 POC System, Roche Diagnostics Corporation, Indianapolis, IA, USA). Biochemical variables were determined in serum using an automated analyzer (Roche Diagnostics Corporation, Indianapolis, IA, USA). Interleukins IL-1β, IL-6, and IL-10, tumor necrosis factor-alpha (TNF-α), and soluble urokinase-type plasminogen activator receptor (suPAR) were determined by an enzyme-linked immunosorbent assay (ELISA) (eBiosciences, San Diego, CA, USA and suPARnostic™, ViroGates, Lyngby, Denmark). The lactate to albumin ratio was calculated by taking into account the molecular weight of lactate (89.07 g) [found in https://pubchem.ncbi.nlm.nih.gov/compound/Lactate, accessed on 8 July 2024] to end up with a dimensionless quantity. It is important to mention that previous studies simply calculate the ratio of lactate in mmol/L to albumin in g/dL or g/L, without the required unit conversions based on the molecular weight of lactate [29,30].

### 2.3. Statistical Analysis

For the assessment of categorical variables, we applied the chi-square test. The Shapiro–Wilk test was used to test the normality hypothesis. Normally distributed variables were expressed as mean ± standard deviation (SD), whilst not normally distributed variables were expressed as median and interquartile range. We performed the *t*-test and paired *t*-test for normally distributed variables and the Mann–Whitney U test and the Wilcoxon matched-pair test for not normally distributed variables. The Spearman correlation coefficients (*r*) were used to determine the association between continuous variables. Receiver Operating Characteristic (ROC) curves were generated to evaluate mortality prediction. The Kaplan–Meier method was used to produce survival curves, while comparisons were performed using the log-rank test. Univariate and multivariate Cox regression analyses were performed to evaluate independent predictors of 28-day mortality. Reported *p*-values were two-sided, with a value of α = 0.05 considered significant. We used the statistical package IBM-SPSS ^®^ version 29 for Windows for the statistical analysis.

## 3. Results

### 3.1. Characteristics of the Study Population

We included 102 critically ill patients with new onset sepsis within 48 h from diagnosis. Sixty patients presented with sepsis at enrollment and forty-two presented with septic shock, according to the SEPSIS-3 diagnostic criteria. Sixty-one cases were medical and forty-one were surgical/trauma according to the diagnosis on ICU admission. The primary site of infection was the lung (*n* = 36), the abdomen (*n* = 24), the blood (*n* = 14), the kidney (*n* = 6), the skin and soft tissues (*n* = 8), the central nervous system (*n* = 12), and the endocardium (*n* = 2). The responsible pathogen was identified in 60 cases as follows: 16 *Acinetobacter* spp., 10 *Pseudomonas* spp., 8 *Klebsiella pneumoniae*, 1 *Escherichia coli*, 1 *Providencia stuartii*, 14 *Staphylococcus* spp., and 10 *Candida species*. Twenty-eight days after inclusion in the study, 72 patients were alive (survivors), and 30 patients were deceased (nonsurvivors). The mortality rate of our study cohort was 29.4%, while patients with sepsis and septic shock presented a mortality rate of 10% and 57%, respectively. The baseline characteristics and laboratory parameters of patients at inclusion in the study and one week later are depicted in Table 1.

### 3.2. Kinetics of LAR According to Severity and Outcome

**Figure 1:**
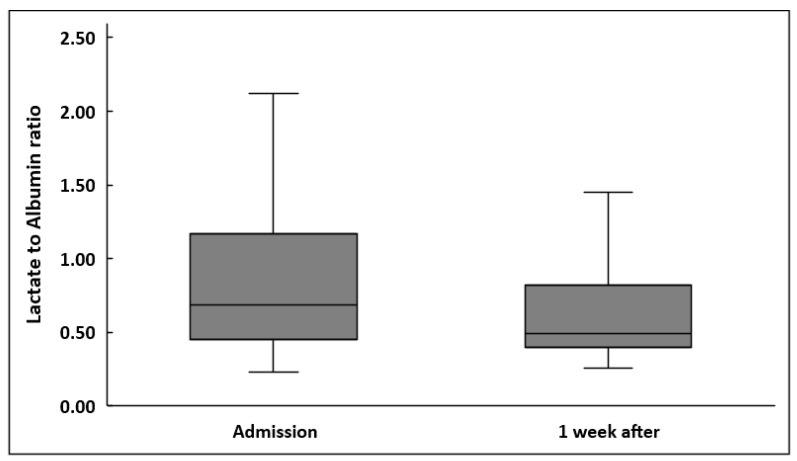
Lactate to albumin ratio upon inclusion in the study and one week after in 102 patients.

LARs were significantly decreased one week after sepsis onset in all patients (0.69 (0.23–3.06) vs. 0.49 (0.26–7.36), *p* < 0.001) (Figure 1). Lactate also decreased significantly (2.1 (1–9) mmol/L vs. 1.3 (0.7–19) mmol/L, *p* < 0.001). However, albumin did not change significantly (2.46 ± 0.59 g/dL vs. 2.41 ± 0.47 g/dL, *p* = 0.27).

**Figure 2:**
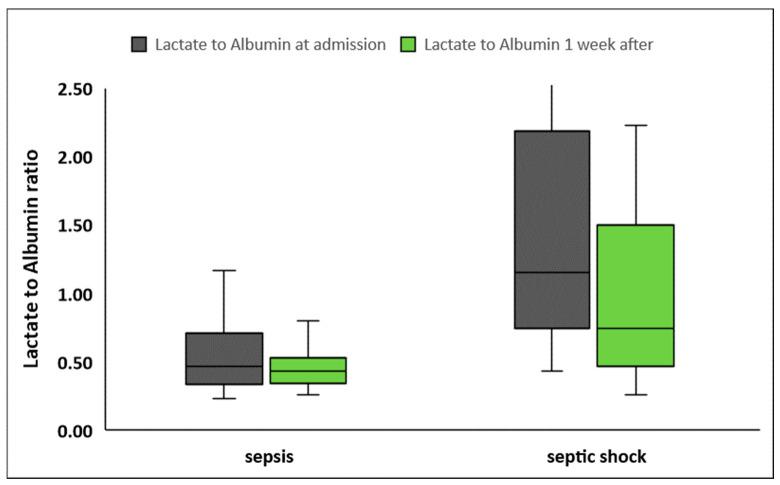
Lactate to albumin ratio upon inclusion in the study and one week later in patients with sepsis (*n* = 60) and septic shock (*n* = 42).

**Figure 3:**
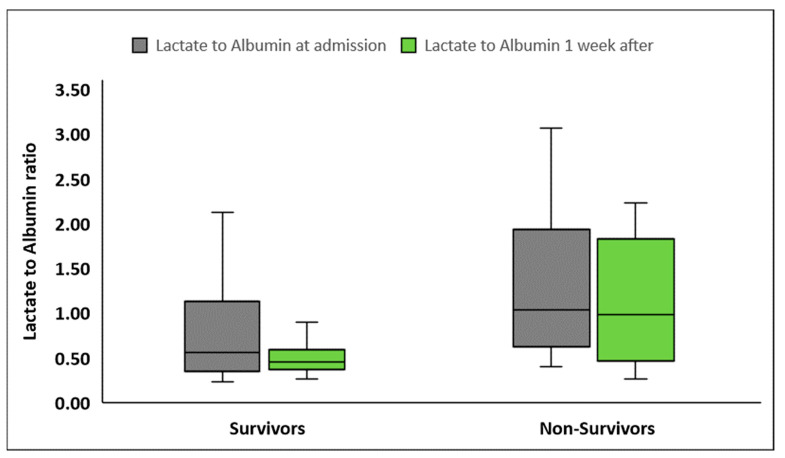
Lactate to albumin ratio upon inclusion in the study and one week later in survivors (*n* = 72) and nonsurvivors (*n* = 30).

LARs were significantly higher in patients presenting with septic shock compared to patients with sepsis, at inclusion in the study as well as one week later (Table 2, Figure 2). LARs were also higher in nonsurvivors compared to survivors, both at sepsis onset and one week later (Table 2, Figure 3). Although LARs decreased significantly in all patients one week after enrollment, LAR differences during the first week were greater in patients with septic shock compared to patients with sepsis (0.20 ± 1.6 vs. 0.12 ± 0.39, *p* < 0.001 respectively). Finally, the decrease in LARs and their percentage changes from baseline did not differ significantly between nonsurvivors and survivors (*p* = 0.88 and *p* = 0.82, respectively).

### 3.3. Correlations of LARs with Other Parameters

**Figure 4:**
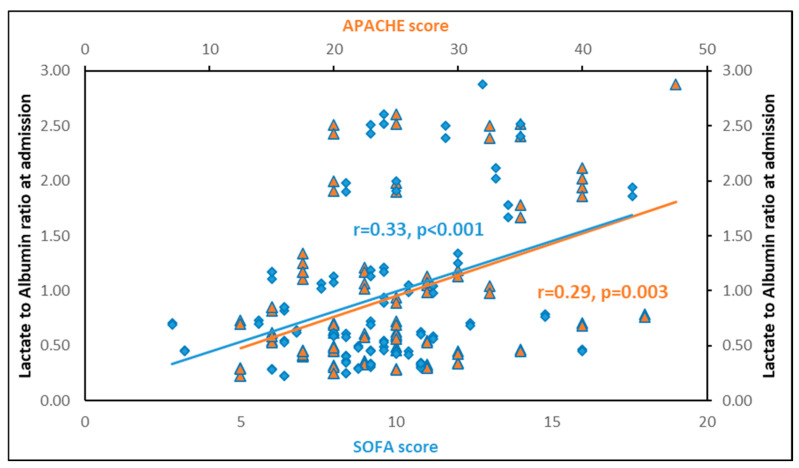
Lactate to albumin ratio upon inclusion in the study is significantly associated with APACHE II (r = 0.29, *p* = 0.003) and SOFA (r = 0.33, *p* < 0.001) scores. In blue: correlation of LAR with SOFA score. In orange: correlation of LAR with APACHE score.

LARs upon inclusion in the study presented significant positive associations with the severity scores APACHE II (*p* = 0.003) and SOFA (*p* < 0.001) (Figure 4). Additionally, LARs were negatively associated with platelets (*p* = 0.01) and total protein (*p* < 0.001), but not with white blood cells. It also showed a significant positive association with creatinine (*p* = 0.01), lactate dehydrogenase (*p* < 0.001), prothrombin time (*p* < 0.01) and activated partial thromboplastin time (*p* < 0.001). Finally, LARs were significantly associated with C-reactive proteins (CRPs) (*p* = 0.002), procalcitonin (*p* < 0.001), IL-6 (*p* = 0.005), IL-10 (*p* = 0.002), and suPAR (*p* = 0.004), but not with IL-1β and TNF-α (Table 3).

### 3.4. LAR and Sepsis Outcome

**Figure 5:**
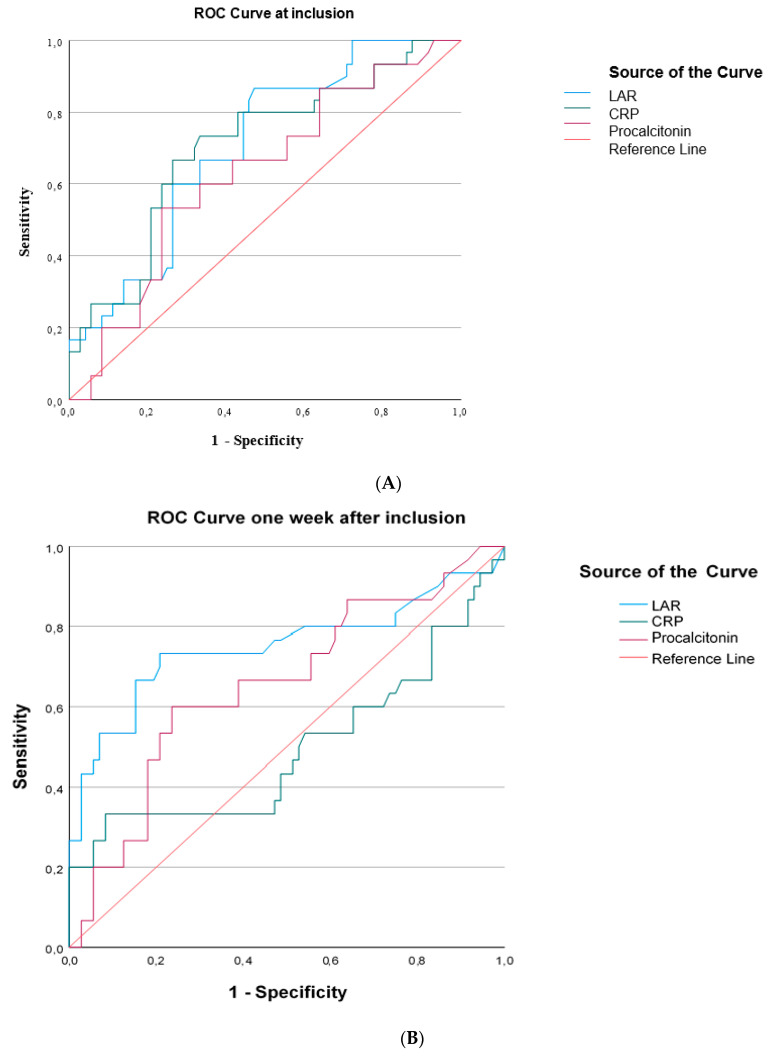

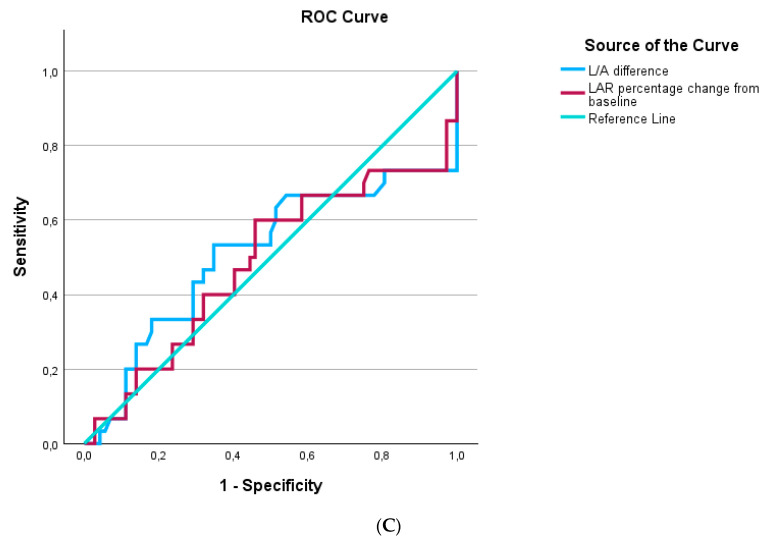
The area under the Receiver Operating Characteristic curve (AUROC) distinguishing survivors from nonsurvivors in 102 patients with sepsis. (**A**) **At inclusion:** LAR, AUROC > 0.706; CRP, AUROC > 0.705; procalcitonin, AUROC > 0.628. (**B**) **One week after inclusion:** LAR, AUROC > 0.750; CRP, AUROC > 0.497; procalcitonin, AUROC > 0.653. (**C**) LAR kinetics expressed as LAR difference, AUROC > 0.51, and LAR percentage change from baseline, AUROC > 0.48.

ROC curves were generated for LAR and inflammatory biomarkers that differed significantly between survivors and nonsurvivors at sepsis onset and one week later. At inclusion in the study, LARs performed similarly to CRPs and procalcitonin in discriminating survivors from nonsurvivors (Figure 5A). One week after inclusion in the study, LARs presented similar discriminating abilities with CRPs and procalcitonin (Figure 5B). LAR kinetics, expressed as the decrease in LARs and LAR percentage change from baseline, could not discriminate survivors from nonsurvivors (Figure 5C).

**Figure 6:**
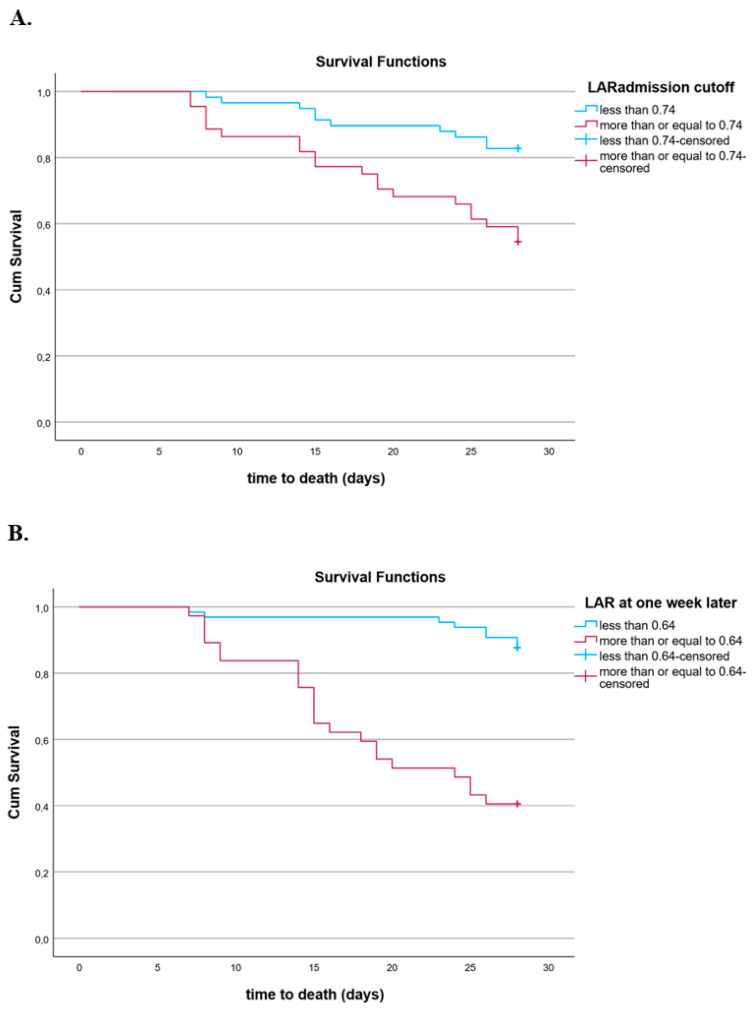
(**A**) Kaplan–Meier estimates of mortality in 102 septic patients based on LARs at inclusion cutoff values obtained via ROC analysis (log-rank test: 9.904, *p* = 0.002). (**B**) Kaplan–Meier estimates of mortality in 102 septic patients based on LAR one week after inclusion cutoff values obtained via ROC analysis (log-rank test: 30.57, *p* < 0.001).

The Kaplan–Meier survival curves demonstrated that patients with a lower LAR at sepsis onset presented a better outcome at 28 days, with the cutoff value of the LAR being 0.74 (Figure 6A). Additionally, patients with a lower LAR one week after inclusion in the study had improved survival at 28 days, with the cutoff value of the LAR estimated at 0.64 (Figure 6B).

Unadjusted Cox regression analyses showed that LARs at sepsis onset (HR: 1.92, 95% C.I. 1.26–2.94, *p* = 0.003) and one week later (HR: 1.37, 95% C.I. 1.16–1.62, *p* < 0.001) were significantly associated with 28-day mortality. Regarding LAR kinetics, neither LAR difference nor LAR percentage change from baseline was associated with 28-day mortality (HR: 0.82, 95% C.I. 0.65–1.03, *p* = 0.08). Furthermore, multivariate Cox regression analyses adjusting for significant variables, showed that higher LAR was an independent predictor of 28-day mortality at sepsis onset (HR 2.27, 95% C.I. 1.01–5.09, *p* = 0.04) and one week later (HR: 4.29, 95% C.I. 1.71–10.78, *p* = 0.002) (Table 4).

Of note, IL-1b, IL-6, IL-10, TNF-a, and suPAR did not significantly change during the first week of sepsis (*p* > 0.05) (Table 1). Our analysis showed that none of the kinetic changes in the inflammatory biomarkers were associated with sepsis outcome (*p* > 0.05). In particular, kinetics of IL-1b (HR: 0.99, 95% C.I. 0.99–1.007, *p* = 0.81), IL-6 (HR: 1.002, 95% C.I. 0.99–1.005, *p* = 0.37), IL-10 (HR: 1.002, 95% C.I. 0.99–1.010, *p* = 0.59), TNF-α (HR: 0.99, 95% C.I. 0.98–1.006, *p* = 0.56), and suPAR (HR: 0.96, 95% C.I. 0.85–1.14, *p* = 0.84) were not associated with 28-day mortality.

## 4. Discussion

In this prospective observational study, we explored the kinetics of LARs in critically ill patients with new onset sepsis or septic shock during the first week from sepsis onset. We found that LARs were significantly lower one week after enrollment compared to baseline in all patients. We also demonstrated that LARs were significantly higher in patients who presented with septic shock compared to patients with sepsis both at inclusion and one week later. LARs at inclusion in the study were positively associated with the severity of sepsis, as reflected by the severity scores APACHE II and SOFA, as well as with important inflammatory biomarkers. In addition, we found that LARs were significantly higher in nonsurvivors compared to survivors at baseline and one week later. Furthermore, we showed that patients with lower LARs at baseline and one week after had a better outcome at 28 days. Finally, we demonstrated that higher LARs at sepsis onset and one week after was an independent predictor of 28-day mortality. However, LAR kinetics was not associated with 28-day mortality.

The LAR has recently attracted attention as an index for prognostic evaluation of critical illness and sepsis. Since the LAR reflects changes in two important prognostic biomarkers of sepsis in opposing directions, it may present a potentially promising prognostic index. Recent evidence from large retrospective studies supports that the LAR may be a useful prognostic biomarker in critical illness and sepsis [19,21,31]. Furthermore, LARs upon admission have been retrospectively evaluated in patients with sepsis presented to the emergency department. In two large studies from Korea, retrospective analysis of data from the multicenter registry of the Korean Shock Society in patients with sepsis showed that LARs on admission predicted 28-day mortality [32,33]. In addition, recent retrospective studies in critically ill patients with sepsis admitted to the ICU have demonstrated that the LAR at admission was an independent predictor of mortality, in line with our findings [23,34,35]. Other retrospective studies also corroborated the prognostic value of LARs in critically ill patients with sepsis [36,37]. Of note, a few studies have indicated that LARs may predict mortality in patients with sepsis-associated kidney or liver injury [19,32,38]. However, the retrospective design of these studies limits the value of their results.

There is only a limited number of prospective studies on the prognostic value of LAR in patients with sepsis. A large prospective study of 939 patients with sepsis presenting to the emergency department evaluated the LAR as a prognostic indicator of hospital mortality with positive results [30]. A recent study of 459 septic patients admitted to the emergency department showed that the LAR on admission was an independent predictor of 30-day mortality, in agreement with our findings [39]. Another study of 160 patients admitted to the ward with sepsis demonstrated that LARs were associated with hospital mortality [29]. There are only two prospective studies in patients with sepsis admitted to the ICU. Wang et al. investigated the LAR in 54 patients with severe sepsis and septic shock and found that the LAR was associated with the severity of sepsis and was a good predictor of multiple organ dysfunction syndrome and mortality [22]. In addition, Shadvor et al. investigated 151 ICU patients with new onset septic shock and found that LAR at 6 h from the onset was associated with mortality, length of ICU stay, and duration of mechanical ventilation [40].

Our findings corroborate the results of previous studies and recent meta-analyses on the association of LARs with the severity of sepsis and their prognostic role [41,42]. However, all previous studies have evaluated LARs on admission, a time point that does not safely denote the onset of sepsis. In contrast, we followed critically ill patients in the ICU and enrolled only patients with new onset sepsis, while other studies enrolled patients on admission without recording the actual onset of sepsis. Additionally, our methodology was carefully designed to outperform previous studies. Specifically, our study is the first to correctly calculate the LAR as a net number, by conversing lactate and albumin to the same units based on the molecular weight of lactate. Finally, this is the first study to investigate the kinetics of LARs during the first week, indicating that the LAR may be a useful index associated with the severity and mortality not only at sepsis onset but also later on in the course of sepsis. Therefore, our study may prove useful in clinical practice since both lactate and albumin are readily measured in septic patients.

The main strengths of our study lie in its prospective design and the careful selection of patients according to specific inclusion and exclusion criteria. Of note, we have excluded patients with preexisting liver disease that may affect the production of albumin as well as lactate clearance. Moreover, we excluded patients receiving albumin- or lactate-containing fluids, which are commonly administered in critically ill patients and are important confounding factors. Finally, we have calculated the LAR as a net number, without units, according to the molecular weight of lactate. Nevertheless, our study has certain limitations as well. It is a single-center study. Therefore, these results may not apply to other critically ill populations with sepsis. However, all patients were treated according to international guidelines. In addition, we have not excluded patients with renal failure, which may have affected lactate and albumin clearance, albeit to a lesser extent than liver disease. Additionally, we have not taken into account the incidence of acute liver and kidney injury/failure during the study, which may have confounded LAR kinetics [43]. Finally, due to the moderate sample size of our study, it was impossible to include all biochemical parameters in the analysis.

## 5. Conclusions

In critically ill patients with new onset sepsis, LARs at sepsis onset as well as one week later are associated with the severity and outcome of sepsis. A higher LAR at sepsis onset and one week later is an independent predictor of 28-day mortality, while LAR kinetics is not associated with mortality. The LAR may be a useful prognostic index in patients with sepsis.

## Figures and Tables

**Table 1:** Baseline characteristics and laboratory variables of patients at inclusion in the study and one week later.

Variables	At Inclusion (*n* = 102)	One Week Later (*n* = 102)	*p*-Value
Demographic variables			
Age ^a^, years	64.7 ± 15.6		
Sex, male, n (%)	57 (55.9)		
BMI ^a^, kg/m^2^	29.9 ± 8.5		
Clinical variables			
APACHE II ^a^	23 ± 7.2		
SOFA ^a^	10 ± 3.3		
Septic shock, n (%)	42 (41.2)		
Death at 28 days, n (%)	30 (29.4)		
Hematologic variables			
Hemoglobin ^a^, g/L	93 ± 20	87 ± 15	**0.002**
White blood cells ^a^ × 10^9^/L	14.1 ± 8.4	11.6 ± 8.4	**0.005**
Platelets ^a^ × 10^9^/L	216.2 ± 118.8	220.5 ± 117.7	0.67
Metabolic variables			
Albumin ^a^, g/dL	2.46 ± 0.59	2.41 ± 0.47	0.27
Lactate ^b^, mmol/L	2.1 (1–9)	1.3 (0.7–19)	**<0.001**
LAR ^b^	0.69 (0.23–3.06)	0.49 (0.26–7.36)	**<0.001**
Creatinine ^a^, μmol/L	123.76 ± 70.72	120.25 ± 107.87	0.13
Total protein ^a^, g/dL	5 ± 0.9	4.9 ± 0.8	0.10
LDH ^a^, U/L	338.6 ± 186.9	324.2 ± 167.1	0.27
Coagulation variables			
Prothrombin time ^a^, s	14.3 ± 4.7	13.6 ± 2.9	0.09
aPTT ^a^, s	38.9 ± 9.4	38.4 ± 9.6	0.47
Fibrinogen ^a^, μmol/L	14.49 ± 5.26	13.79 ± 5.28	0.19
Inflammatory variables			
CRP ^b^, nmol/L	1257 (67–4105)	641 (79–2686)	**<0.001**
Procalcitonin ^b^, μg/L	0.9 (0.1–100)	0.6 (0.1–82.6)	**<0.001**
IL-1β ^b^, ng/L	5.9 (5.9–206)	10.5 (5.9–499)	0.26
IL-6 ^b^ ng/L	27.4 (6–444)	24.2 (4.6–487)	0.46
IL-10 ^b^, ng/L	5 (5–300)	5 (5–300)	0.51
TNF-α ^b^, ng/L	6 (6–337)	6 (6–812)	0.34
suPAR ^b^, μg/L	13 (2.1–16.8)	11.6 (2.6–16.8)	0.8

Abbreviations: APACHE II, acute physiology and chronic health evaluation score; aPTT, activated partial prothrombin time; BMI, body mass index; CRP, C-reactive protein; IL, interleukin; LAR, lactate to albumin ratio; LDH, lactate dehydrogenase; SOFA, sequential organ failure assessment score; suPAR, soluble urokinase-type plasminogen activator receptor; TNF-α, tumor necrosis factor-alpha. Significant differences are highlighted in bold. ^a^ Mean ± SD, ^b^ Median, range.

**Table 2:** Albumin, lactate, and lactate to albumin ratio in patients with sepsis and septic shock as well as in survivors and nonsurvivors at inclusion in the study and one week later.

		At Inclusion		One Week after Inclusion		
**Variables**	**Sepsis (*n* = 60)**	**Septic Shock (*n* = 42)**	***p*-Value**	**Sepsis (*n* = 60)**	**Septic Shock (*n* = 42)**	***p*-Value**
Albumin ^a^, g/dL	2.6 ± 0.56	2.26 ± 0.57	0.004	2.51 ± 0.48	2.25 ± 0.42	0.005
Lactate ^b^, mmol/L	1.2 (1–5)	2.4 (2.1–9)	<0.001	1 (1–2.7)	1.9 (0.7–19)	<0.001
LAR ^b^	0.47 (0.23–1.98)	1.15 (0.43–3.06)	<0.001	0.43 (0.26–1.10)	0.74 (0.26–7.36)	<0.001
	**Survivors (*n* = 72)**	**Nonsurvivors (*n* = 30)**	***p*-Value**	**Survivors (*n* = 72)**	**Nonsurvivors (*n* = 30)**	***p*-Value**
Albumin ^a^, g/dL	2.51 ± 0.61	2.34 ± 0.52	0.187	2.47 ± 0.5	2.26 ± 0.37	0.04
Lactate ^b^, mmol/L	1.45 (1–9)	2.3 (1.2–8.5)	0.01	1.2 (1–3.5)	2.1 (0.74–19)	<0.001
LAR ^b^	0.55 (0.23–2.51)	1.03 (0.40–3.06)	0.001	0.45 (0.26–1.73)	0.98 (0.26–7.36)	<0.001

Abbreviations: LAR, lactate to albumin ratio. ^a^ Mean ± SD, ^b^ Median, range.

**Table 3:** Spearman correlation coefficients (*r*) of lactate to albumin ratio with severity scores and laboratory biomarkers in septic patients at inclusion in the study (*n* = 102).

Variables	*r*	*p*
Clinical scoring		
APACHE II	**0.29**	**0.003**
SOFA	**0.33**	**<0.001**
Hematologic variables		
White blood cells	0.03	0.77
Platelets	**−0.25**	**0.01**
Metabolic variables		
Total protein	**−0.34**	**<0.001**
Creatinine	**0.24**	**0.01**
LDH	**0.33**	**<0.001**
Coagulation variables		
Prothrombin time	**0.26**	**<0.01**
aPTT	**0.40**	**<0.001**
Fibrinogen	−0.07	0.47
Inflammatory variables		
CRP	**0.29**	**0.002**
Procalcitonin	**0.47**	**<0.001**
IL-1β	0.02	0.87
IL-6	**0.28**	**0.005**
IL-10	**0.30**	**0.002**
TNF-α	−0.018	0.85
suPAR	**0.28**	**0.004**

Abbreviations: APACHE II, acute physiology and chronic health evaluation score; aPTT, activated partial thromboplastin time; CRP, C-reactive protein; IL, interleukin; LDH, lactate dehydrogenase; SOFA, sequential organ failure assessment score; suPAR, soluble urokinase-type plasminogen activator receptor; TNF-α, tumor necrosis factor-alpha. Significant correlations are highlighted in bold.

**Table 4:** Multivariate Cox regression analysis for the independent predictors of mortality (expressed as quartiles) adjusting for APACHE II score in 102 patients with sepsis.

	b	SE_b_	Wald	df	*p*-Value	HR	95% for C.I.
**Independent Predictors at Inclusion**							
LAR	0.819	0.413	3.93	1	0.04	2.27	1.01–5.09
CRP	0.271	0.191	2.02	1	0.15	1.31	0.90–1.90
IL-6	0.061	0.171	0.12	1	0.72	1.06	0.76–1.48
APACHE II	0.349	0.194	3.23	1	0.07	1.42	0.97–2.07
**Independent Predictors One Week after Inclusion**							
LAR	1.458	0.47	9.64	1	0.002	4.29	1.71–10.78
CRP	−0.054	0.17	0.09	1	0.76	0.95	0.67–1.33
IL-6	0.34	0.19	2.95	1	0.08	1.40	0.95–2.07
APACHE II	0.437	0.23	3.75	1	0.05	1.55	1.00–2.07

Abbreviations: APACHE II, acute physiology and chronic health evaluation score; b, regression coefficient; C.I., confidence interval; CRP, C-reactive protein; df, degree of freedom; HR, hazard ratio; IL-6, interleukin 6; LAR, lactate to albumin ratio; SEb, standard error of b.

## Data Availability

Data to support the findings of this study are available upon reasonable request.

## Abbreviations

APACHE, acute physiology and chronic health evaluation; aPTT, activated partial thromboplastin time; AUROC, area under the Receiver Operating Characteristic curve; C.I., confidence interval; CRP, C-reactive protein; ELISA, enzyme-linked immunosorbent assay; HR, hazard ratio; ICU, Intensive Care Unit; IL, interleukin; LAR, lactate to albumin ratio; LDH, lactate dehydrogenase; ROC, Receiver Operating Characteristic; SD, standard deviation; SOFA, sequential organ failure assessment; suPAR, soluble urokinase-type plasminogen activator receptor; TNF-α, tumor necrosis factor-alpha.
